# Heterogeneity of Mesenchymal Markers Expression—Molecular Profiles of Cancer Cells Disseminated by Lymphatic and Hematogenous Routes in Breast Cancer

**DOI:** 10.3390/cancers5041485

**Published:** 2013-11-08

**Authors:** Aleksandra Markiewicz, Magdalena Książkiewicz, Barbara Seroczyńska, Jarosław Skokowski, Jolanta Szade, Marzena Wełnicka-Jaśkiewicz, Anna J. Zaczek

**Affiliations:** 1 Department of Medical Biotechnology, Intercollegiate Faculty of Biotechnology, University of Gdańsk and Medical University of Gdańsk, Gdańsk 80-211, Poland; E-Mails: aleksandra.markiewicz@biotech.ug.edu.pl (A.M.); ksiazkiewiczmagdalena@gmail.com (M.K.); 2 Postgraduate School of Molecular Medicine, Medical University of Warsaw, Warsaw 02-091, Poland; 3 Bank of Frozen Tissues and Genetic Specimens, Department of Medical Laboratory Diagnostics, Medical University of Gdańsk, Gdańsk 80-211, Poland; E-Mails: bastrzel@gumed.edu.pl (B.S.); jskokowski@gumed.edu.pl (J.S.); 4 Department of Surgical Oncology, Medical University of Gdańsk, Gdańsk 80-214, Poland; 5 Department of Pathomorphology, Medical University of Gdańsk, Gdańsk 80-214 Poland; E-Mail: jszade@gumed.edu.pl; 6 Department of Oncology and Radiotherapy, Medical University of Gdańsk, Gdańsk 80-211, Poland; E-Mail: mwelj@gumed.edu.pl

**Keywords:** breast cancer, lymph node metastasis, circulating tumor cells, metastasis, cancer dissemination, epithelial-mesenchymal transition

## Abstract

Breast cancers can metastasize via hematogenous and lymphatic routes, however in some patients only one type of metastases are detected, suggesting a certain proclivity in metastatic patterns. Since epithelial-mesenchymal transition (EMT) plays an important role in cancer dissemination it would be worthwhile to find if a specific profile of EMT gene expression exists that is related to either lymphatic or hematogenous dissemination. Our study aimed at evaluating gene expression profile of EMT-related markers in primary tumors (PT) and correlated them with the pattern of metastatic spread. From 99 early breast cancer patients peripheral blood samples (N = 99), matched PT (N = 47) and lymph node metastases (LNM; N = 22) were collected. Expression of *TWIST1*, *SNAI1*, *SNAI2* and *VIM* was analyzed in those samples. Additionally expression of *CK19*, *MGB1* and *HER2* was measured in CTCs-enriched blood fractions (CTCs-EBF). Results were correlated with each other and with clinico-pathological data of the patients. Results show that the mesenchymal phenotype of CTCs-EBF correlated with poor clinico-pathological characteristics of the patients. Additionally, PT shared more similarities with LNM than with CTCs-EBF. Nevertheless, LNM showed increased expression of EMT-related markers than PT; and EMT itself in PT did not seem to be necessary for lymphatic dissemination.

## 1. Introduction

Metastatic spread still remains the main cause of deaths in breast cancer. There are two main routes of cancer cells dissemination in breast cancer: lymphatic and hematogenous.

Axillary lymph node status is one of the most important prognostic factors for survival in breast cancer [1]. However, clinical studies have shown that approximately 25% of patients with negative lymph node status still develop systemic recurrence and die of the disease [2,3,4]. This might suggest that hematogenous spread occurs in a substantial number of patients and is independent of lymphatic involvement.

The hematogenous route of dissemination can be manifested as disseminated cells in bone marrow (DTCs) or circulating tumor cells (CTCs) in the bloodstream of cancer patients. In the pooled analysis of 4,703 patients with operable breast cancer DTCs have been shown to be a negative prognostic factor [5], like CTCs analyzed in a recent meta-analysis [6]. The number of CTCs carries prognostic information in both early [7,8] and metastatic breast cancer [9,10,11]. Also, CTCs detected via PCR-based methods have been shown to be associated with poor prognosis in a number of studies [12,13], summarized by Zhao [14]. CTCs may constitute seeds for subsequent growth of metastasis in distant organs, according to Paget’s “seed and soil hypothesis” [15]. Nevertheless, they represent a heterogeneous population of tumor cells and only some of them are capable of developing metastasis. 

Epithelial-mesenchymal transition (EMT) has been found to be crucial in cancer dissemination, endowing tumor cells with metastatic and cancer stem cell properties [16,17]. It is characterized by downregulation of epithelial markers (e.g., cytokeratin 8, 18, 19, E-cadherin, claudins, occludins) and upregulation of mesenchymal markers (e.g., *N*-cadherin, fibronectin, vimentin, tenascin C) [18], what results in numerous phenotypic changes such as the loss of cell-cell adhesion and cell polarity, and the acquisition of migratory and invasive properties [19,20]. TWIST1, SNAI1 and SNAI2 are transcription factors governing EMT [19]. Their increased expression in primary tumor (PT) has been associated with poor prognostic clinico-pathological features and worse outcome in breast and other cancers [21,22,23]. Also increased expression of *TWIST1* and *SNAI1* in lymph node metastasis (LNM) of early breast cancer patients, reported previously by our group, conferred worse prognosis, confirming the correlation of EMT with aggressive disease behavior [24]. EMT seems to be involved in metastatic potential of CTCs [25,26,27]. Mesenchymal markers on CTCs have been detected in numerous studies [28,29,30], they occurred more frequently in metastatic compared to early breast cancer [26]. Mesenchymal CTCs were associated with disease progression [30] and allowed more accurate prediction of worse prognosis than the expression of epithelial markers alone [31]. 

Correlation of CTCs presence in blood with lymphatic spread is still controversial. Some studies have revealed that CTCs might be shed by primary tumors independently of lymph node status [7,32,33,34,35], while other presented strong correlations between CTCs and lymph node involvement [36], compiled also by recent meta-analysis [14]. 

It has been hypothesized that some tumors, like breast cancer [37,38,39], may spread preferentially via lymphatic pathways to lymph nodes, whereas others, e.g., sarcomas [37,39], may metastasize directly via a hematogenous route at an early stage. These pathways are governed by the biological characteristics of the primary tumor (PT), complemented by factors in the metastatic host environment [40]. A preferential metastasis route might result from differences in structure and accessibility of lymphatic and hematogenous vasculature in the primary tumor [41]. We have hypothesized that tumor cells disseminated via lymphatic or hematogenous route show different levels of EMT activation as a result of varying changes cancer cells need to adapt in order to enter and survive in those circulatory systems.

In the current study we have prospectively examined patterns of metastatic spread to lymph nodes and peripheral blood in a group of patients with early breast cancer. We have evaluated gene expression patterns of EMT-related markers in PT and correlated them with the pattern of metastatic spread for the individual tumors as determined by the presence or absence of lymph node metastases (LNM) or CTCs. We also compared EMT-related gene expression pattern in PT with expression patterns in LNM and CTCs enriched blood fractions (CTCs-EBF). The focus was also put on comparison of epithelial and mesenchymal CTCs phenotypes in relation to clinical data.

## 2. Results

### 2.1. Occurrence of CTCs and LNM

CTCs enrichment and functional RNA isolation was successful in 90% (89/99) of cases. No CTCs-EBF from healthy controls had detected expression of *TWIST1*, *SNAI1*, *SNAI2*, *HER2*, *CK19* and *MGB1.* Therefore every expression of these genes in patient’s samples was considered positive. Expression of *VIM* was found in nine healthy controls—median *VIM* expression level 11.4 (range 0–30.7). The maximal expression level found in CTC-EBF of healthy donors—30.7 was the threshold level above which CTCs-EBF of breast cancer patients were considered positive. Epithelial positive CTCs-EBF defined as *CK19+* occurred in 27% (24/89) of cases. Positive result for *MGB1* and *HER2* was found in 11.5% (10/87) and 36% (31/87) of cases, respectively. 

Multimarker-based approach for *CK19+* and/or *MGB1+* gave 34% (30/89) detection rate, for *CK19+* and/or *MGB1+* and/or *HER2+* 55% (49/89) (Table 1). 

Mesenchymal positive CTCs-EBF, defined as *VIM+* was found in 20% (18/89) of cases, *TWIST1* was positive in 2% (2/87), *SNAI1* in 8% (7/87) of cases. There was no positive result for *SNAI2*, according to defined criteria. Mesenchymal positive CTCs-EBF based on multimarker definition (*VIM+* and/or *SNAI1+* and/or *TWIST1+*) occurred in 25% (22/87) of cases. 

In the CTC+ group (*CK19+* and/or *MGB1+* and/or *HER2+*) the expression rates of mesenchymal markers were higher than in CTC− group (28% *vs.* 10% for *VIM*, 4% *vs.* 0% for *TWIST1* and 15% *vs.* 0% for *SNAI1*). Altogether positive mesenchymal markers status occurred in 38% of CTC+ cases compared to 10% CTC− (*p* = 0.002; Table 1).

**Table 1:** Correlation between CTCs detection status and mesenchymal markers expression status.

Marker	Expression status	CTC− N (%)	CTC+ * N (%)	*p*
Mesenchymal markers	Negative	36 (90)	29 (62)	**0.002**
	Positive	4 (10)	18 (38)	
*VIM*	Negative	36 (90)	35 (72)	**0.03**
	Positive	4 (10)	14 (28)	
*TWIST1*	Negative	40 (100)	45 (96)	0.19
	Positive	0 (0)	2 (4)	
*SNAI1*	Negative	40 (100)	40 (85)	**0.01**
	Positive	0 (0)	7 (15)	

* CTC positivity defined as *CK19+* and/or *MGB1+* and/or *HER2+.* Statistically significant *p* values in bold.

Lymph node involvement was observed in 51% (45/89) of cases. It positively correlated with both CTCs detected by multimarker method (defined as *CK19+* and/or *MGB1+* in a double marker assay; or *CK19+* and/or *MGB1+* and/or *HER2+* in a triple marker assay) and mesenchymal positive (*VIM+*) CTCs-EBF (*p* = 0.009, *p* = 0.008, *p* = 0.04, respectively), but not with epithelial positive (*CK19+*) CTCs-EBF (*p* = 0.17; Table 2). We have not observed any statistically significant correlation between expression of *VIM*, *TWIST1* or *SNAI1* (analyzed together or separately) in CTCs-EBF and histological type of the tumor (ductal *vs.* lobular, *p* = 0.48) or molecular subtype of the primary tumor (*p* = 0.93, data not shown).

**Table 2:** Correlation between CTCs-EBF gene expression status (applying different phenotypes classification) and lymph node status.

CTCs characteristics	status	Lymph node status ^#^			*p* value
		N	Negative (%)	Positive (%)	
		89 (100)	44 (50)	45 (51)	
*CK19*	Negative	65 (73)	35 (80)	30 (69)	0.17
	Positive	24 (27)	9 (20)	15 (33)	
*CK19/MGB1*	Negative	59 (66)	35 (80)	24 (53)	**0.009**
	Positive	30 (34)	9 (20)	21 (47)	
*CK19/MGB1/HER2*	Negative	40 (45)	26 (59)	14 (31)	**0.008**
	Positive	49 (55)	18 (41)	31 (69)	
*VIM*	Negative	71 (80)	39 (89)	32 (71)	**0.04**
	Positive	18 (20)	5 (11)	13 (29)	
VIM/SNAI1/TWIST1 *	Negative	65 (75)	36 (84)	29 (66)	0.056
	Positive	22 (25)	7 (16)	15 (34)	

* For *TWIST1* and *SNAI1* 87 RT-qPCR results were available. For two samples the reaction was unsuccessful. ^#^ The percentages calculated for columns. Statistically significant *p* values in bold.

### 2.2. Different CTCs-EBF Phenotypes and Clinical Characteristics

Epithelial phenotype of CTCs-EBF based on the identification of *CK19* did not correlate with any clinical data, while mesenchymal positive CTCs-EBF phenotype was more frequent in higher T stage tumors (*p* = 0.0003). Lymph node positive status was observed more frequently in higher T stage (*p* = 0.008) and higher grade tumors (*p* = 0.01; Table 3).

**Table 3:** Basic clinical characteristics of PT in relation to CTCs-EBF epithelial and mesenchymal phenotype and LNM.

PT	CTCs epithelial phenotype			CTCs mesenchymal phenotype			LN status		
	*CK19-*	*CK19+*	*p*	*VIM−*	*VIM+*	*p*	−	+	*p*
N	65	24		71	18		44	55	
Age [median]	63	59	0.2	61.5	67.5	0.35	62	60.5	0.64
T									
1-2	57 (89)	24 (100)	0.09	69 (97)	12 (71)	**0.0003**	44 (100)	46 (85)	**0.008**
3-4	7 (11)	0 (0)		2 (3)	5 (29)		0 (0)	8 (15)	
G									
1-2	42 (66)	16 (67)	0.93	48 (70)	10 (56)	0.29	34 (77)	29 (54)	**0.01**
3	22 (34)	8 (33)		21 (30)	8 (44)		10 (23)	25 (46)	
Hormone receptor status									
Negative	12 (18)	5 (21)	0.8	15 (21)	2 (11)	0.33	7 (16)	12 (22)	0.46
ER and/or PgR positive	53 (82)	19 (79)		56 (79)	16 (89)		37 (84)	43 (78)	
HER2 status									
Negative	48 (76)	19 (79)	0.68	53 (75)	14 (78)	0.85	35 (81)	36 (67)	0.1
Positive	16 (24)	5 (21)		17 (25)	4 (22)		8 (19)	18 (33)	

Statistically significant *p* values in bold.

### 2.3. Primary Tumor Characteristics in Relation to CTCs-EBF Phenotype and LNM

Gene expression level of examined EMT-related markers: *TWIST1*, *SNAI1*, *SNAI2* and *VIM* in PT did not correlate with hematogenous spread expressed as positivity either for CTCs-EBF epithelial or mesenchymal phenotype. *SNAI1* and *VIM* negative status in PT correlated with positive LN status (*p* = 0.03 and *p* = 0.05, respectively; Table 4).

### 2.4. Comparison of Gene Expression Levels in Paired PT *vs.* LNM and Paired PT *vs*. CTCs-EBF

**Figure 1:**
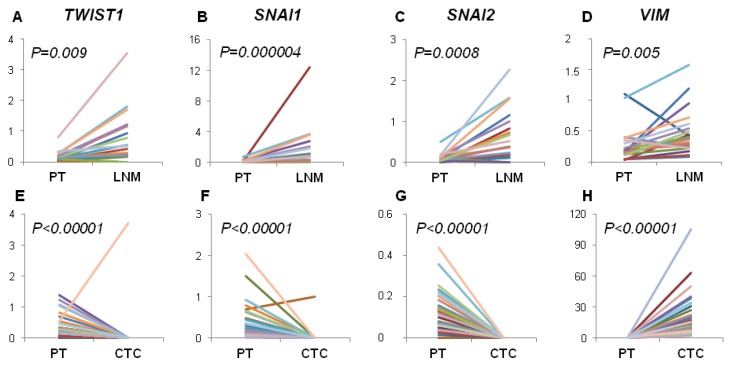
Comparison of gene expression levels in paired PT *vs.* LNM and paired PT *vs.* CTCs-EBF. Figure presents changes in relative gene expression level of *TWIST1*, *SNAI1*, *SNAI2* and *VIM* in PT compared to LNM (**A**–**D**) and PT compared to CTCs (**E**–**H**). Each line represent the results for one patient. Correlations calculated by Mann-Whitney test. Abbreviations: PT—primary tumor, LNM—lymph node metastasis, CTC—CTCs-enriched blood fraction.

Gene expression level of all examined markers was significantly increased in LNM compared to PT when paired samples were examined. Gene expression level of *TWIST1*, *SNAI1* and *SNAI2* was significantly decreased in CTCs-EBF compared to PT, while the level of *VIM* was increased in CTCs-EBF (Figure 1). 

**Table 4:** Expression of mesenchymal markers in PT in relation to CTCs-EBF epithelial and mesenchymal phenotype and LN status.

PT	CTCs epithelial phenotype			CTCs mesenchymal phenotype			LN status		
	*CK19-*	*CK19+*	*p*	*VIM-*	*VIM+*	*p*	−	+	*p*
N	26	11		29	8		21	26	
*TWIST1*									
negative	11 (42)	4 (36)	0.74	13 (45)	2 (25)	0.31	9 (43)	14 (54)	0.45
positive	15 (58)	7 (64)		16 (55)	6 (75)		12 (57)	12 (46)	
*SNAI1*									
negative	11 (42)	6 (55)	0.5	12 (41)	5 (63)	0.29	7 (33)	17 (65)	**0.03**
positive	15 (58)	5 (45)		17 (59)	3 (37)		14 (67)	9 (35)	
*SNAI2*									
negative	11 (42)	5 (45)	0.86	14 (48)	2 (25)	0.24	9 (43)	14 (54)	0.45
positive	15 (58)	6 (55)		15 (52)	6 (75)		12 (57)	12 (46)	
*VIM*									
negative	10 (38)	8 (73)	0.06	15 (52)	3 (37)	0.48	7 (33)	16 (62)	**0.05**
positive	16 (62)	3 (27)		14 (48)	5 (63)		14 (67)	10 (38)	

Statistically significant *p* values in bold.

### 2.5. Correlations between Gene Expression Levels in PT, LNM and CTCs

Correlations between *TWIST1*, *SNAI1*, *SNAI2* and *VIM* gene expression levels in PT and LNM were statistically significant, while there were no such correlations observed for PT and CTCs-EBF. Moreover, the difference in correlations strength between PT and LNM *vs.* PT and CTCs-EBF was significant for *TWIST1* and *SNAI1* (Table 5). 

**Table 5:** Correlations between gene expression levels in PT, LNM and CTCs-EBF.

Marker	PT and LNM			PT and CTCs			*p* *
	N	Rs	*p*	N	Rs	*p*	
*TWIST1*	20	0.77	**0.00007**	36	0.17	0.32	**0.006**
*SNAI1*	20	0.78	**0.0002**	36	0.22	0.2	**0.02**
*SNAI2*	20	0.64	**0.002**	36	X	X	X
*VIM*	20	0.43	0.057	37	0.09	0.57	0.2

* determined by test of differences that compared R_s_ values in both subgroups. Statistically significant *p* values in bold.

### 2.6. Conversion of Biomarkers Status between PT *vs.* LNM and PT *vs.* CTCs-EBF

Conversion rate of examined markers status between paired PT and LNM ranged from 15% for *TWIST1* to 40% for *SNAI1*. *TWIST1* expression between PT and LNM showed the highest concordance level. Mostly occurred conversions from negative EMT-related marker in PT to positive in LNM (Table 6). 

Conversion rate of examined markers status between paired PT and CTCs-EBF was higher and ranged from 46% for *VIM* to 58% for *TWIST1* and *SNAI2*. The concordance of expression between PT and CTCs-EBF was very low for all examined markers (Cohen’s kappa from 0 for *SNAI2* to 0.23 for *SNAI1*). Moreover, for *TWIST1*, *SNAI1* and *SNAI2* occurred only switch from positive in PT to negative in CTCs (Table 7).

**Table 6:** Biomarkers’ conversion rate between paired PT and LNM. Conversion described as the number (percentage) of discordant cases and kappa coefficient of concordance.

Marker	N	Positive in PT	Positive in LNM	Conversion rate PT➔LNM			
		N (%)	N (%)	(−)➔ (+) N (%)	(+)➔ (−) N (%)	N (%)	kappa coefficient (95% CI)
*TWIST1*	20	7 (35)	10 (50)	3 (15)	0 (0)	3 (15)	0.7 (0.4–0.99)
*SNAI1*	20	5 (25)	11 (55)	7 (35)	1 (5)	8 (40)	0.23 (−0.1–0.58)
*SNAI2*	20	9 (45)	12 (60)	4 (20)	1 (5)	5 (25)	0.51 (0.15–0.87)
*VIM*	20	8 (40)	11 (55)	5 (25)	2 (10)	7 (35)	0.31 (−0.08–0.71)

CI: confidence interval.

**Table 7:** Biomarkers’ conversion rate between paired PT and CTCs-EBF. Conversion described as the number (percentage) of discordant cases and kappa coefficient of concordance.

Marker	N	Positive in PT	Positive in CTCs	Conversion rate PT➔CTCs			
		N (%)	N (%)	(−)➔ (+) N (%)	(+)➔ (−) N (%)	N (%)	kappa coefficient (95% CI)
*TWIST1*	36	22 (61)	1 (3)	0 (0)	21 (58)	21 (58)	0.036 (−0.035–0.1)
*SNAI1*	36	20 (55)	1 (3)	0 (0)	18 (50)	18 (50)	0.23 (−0.047–0.15)
*SNAI2*	36	21 (58)	0 (0)	0 (0)	21 (58)	21 (58)	0.00 (0–0)
*VIM*	37	19 (51)	8 (22)	3 (8)	14 (38)	17 (46)	0.095 (−0.16–0.35)

CI: confidence interval.

## 3. Discussion

Cancer dissemination and metastasis formation remain the most intensively investigated issues in cancer research. Recent findings suggested that distinct lymphatic and hematogenous metastatic pathways exist in early breast cancer and that these pathways are governed by specific biological markers [40]. Since EMT was shown to play critical role in cancer dissemination, we focused on gene expression profiles related to lymphatic and hematogenous dissemination. We therefore analyzed the expression of mesenchymal markers connected with EMT activation (*VIM*, *TWIST1*, *SNAI1* and *SNAI2*) in PT, CTCs-EBF and LNM of breast cancer patients.

First, we tried to elucidate if gene expression profile of EMT-related markers in PT correlates with the pattern of metastatic spread. We have found that examined profile of PT did not correlate with hematogenous spread expressed as positivity either for CTCs epithelial (*CK19+*) or mesenchymal (*VIM+*) phenotype or *CK19+* and/or *MGB1*+ and/or *HER2*+ (data not shown). Interestingly, *SNAI1* and *VIM* negative status in PT correlated with lymph node involvement, suggesting that EMT was not involved in lymphatic dissemination. The anatomy of lymphatic vessels/system might make it easier for tumor cells to metastasize to lymph nodes. Lymphatic capillaries lack tight junctions that are present in blood vessels and are not surrounded by pericytes [42]. It was also shown that lymphatic endothelial cells might facilitate intravasation of tumor cells [43] and tumor cells can be transported to lymph nodes by mechanical forces arising from intratumoral interstitial fluid pressure [44]. Permeability of lymphatic vessels could also allow for passage of E-cadherin connected cells clusters [41,45]. If fewer restrains are put on tumor cells metastasizing via the lymphatic route it is conceivable that these cells do not need to acquire special abilities to metastasize to the lymph nodes. Whereas more severe obstacles that tumor cells encounter during hematogenous dissemination force them to acquire additional properties like motility, induced by EMT. Following this reasoning, the study of Giampieri *et al.* [46] showed that most tumor cells which had arrived in lymph nodes present collective migration phenotype. Moreover, these cells are incapable of forming hematogenous metastases [46], whereas cells exhibiting single migration pattern (like those after EMT) are. Hematogenous metastases were also reduced by inhibiting TGF-β signaling, known for induction of EMT. Interestingly, inhibiting TGF-β signaling did not affect lymphatic metastases as visualized by the presence of collectively invading cancer cells in lymph nodes. These observations support our results, showing that EMT in PT is not required for lymph nodes invasion. 

Contrary, we observed increased expression of EMT-related markers in LNM compared to PT and their frequent status conversion rate (15%–35%) from negative in PT to positive in LNM. This finding would indicate that EMT in lymph nodes is more effectively activated than in PT, thus lymph nodes might serve as tumor cells conditioning centers, at which selection of more malignant phenotypes takes place. Our previous results also showed that spread to lymph nodes is not related to EMT process in primary tumor but high expression of *TWIST1* and *SNAI1* in LNM, as well as negative-to-positive conversion of *SNAI1* confer worse prognosis, confirming the correlation of EMT with aggressive disease behavior [24].

On the other hand, *TWIST1*, *SNAI1* and *SNAI2* gene expression level was significantly decreased in CTCs-EBF compared to PT, while the level of *VIM* was increased in CTCs-EBF. It should be kept in mind that EMT might not be the only process involved in tumor cells dissemination. Recent report by Marinari *et al.* showed that epithelial cells can detach from the epithelial layer in a process of delamination [47]. This could explain presence of epithelial cancer cells in blood. Although we did not observe a direct correlation between expression of EMT-related markers in PT and CTCs detection rate it should be noted that *TWIST1*, *SNAI1*, *SNAI2*, being transcription factors, can be expressed at low levels what hampers their detection in rare CTCs and in isolated sections of PT due to time and space-limited activation of EMT process. This could also explain the occurrence of switch in gene expression status from positive in PT to negative in CTC-EBF in case of *TWIST1*, *SNAI1* and *SNAI2.* Nevertheless, elevated expression of *VIM*, but not *TWIST1* and *SNAI1*, was observed in TGF-β-treated, motile breast cancer cells in the study of Giampieri *et al*. [46]. 

Moreover, we noticed that the profile of analyzed gene expression was more conserved between PT and LNM than between PT and CTCs-EBF. This could reflect higher histological similarity of solid tumors and metastases versus solid tumors and CTCs present in blood. However, analysis of single CTCs would be required to confirm this finding.

PCR was demonstrated as the most sensitive technique for detecting CTCs [48,49]. Also, using negative selection with anti-CD45 antibodies that allows for epithelial markers-independent CTCs enrichment, is especially important if CTCs with mesenchymal phenotype are to be captured. As shown by Mego *et al*. both AdnaTest^TM^ and CellSearch^®^ are unable to isolate cells that completed EMT [35] as EpCam may be lost due to EMT [50]. Therefore, we applied negative selection of CTCs and PCR based approach to reliably examine EMT-related markers. 

In our study we have found epithelial CTCs-EBF defined as *CK19+* in 27% of cases, what is in agreement with previous PCR-based studies of early breast cancer patients exploiting CK19 as CTCs marker [13,51,52,53]. Analyzing expression of a panel of different cytokeratins could increase CTCs detection rate, however validity of this approach remains to be determined. As shown by Joosse *et al*. [54] breast cancer cells express various cytokeratins, which can be affected by their molecular subtype. Using cocktail of antibodies against cytokeratins they increased CTCs detection rate in metastatic breast cancer patients. Future research detecting CTCs using PCR based methods should consider using multiple cytokeratins detection in single- or multiplex assays. Multimarker-based detection of CTCs was reported to give higher sensitivity [32,48,55,56,57]. Like others have reported [48,56], a multimarker approach based on addition of *MGB1* alone or together with *HER2* gave higher detection rates in our study (34 and 55%, respectively). Mesenchymal phenotype of CTCs-EBF defined as *VIM+* was observed in 22% of cases, defined as *VIM+* and/or *SNAIL+* and/or *TWIST1+* in 25%, what also remains concordant with reported wide range of detection rate of mesenchymal CTCs 15%–77% [26,28,31,35,58].

The mesenchymal phenotype of CTCs was found to correlate with more aggressive tumor characteristics [25,26] and disease progression [30]; it also occurred more frequently in metastatic compared to early breast cancer [26,30]. Analysis of mesenchymal markers in CTCs allowed more accurate prediction of worse prognosis than the expression of epithelial markers alone [31]. In patients with primary breast cancer the overexpression of EMT-inducing transcription factors (*TWIST1*, *SNAIL1*, *SLUG*, *ZEB1*, and *FOXC2*) was more frequently detected in those who received neoadjuvant therapies, than in those who did not, which suggests that neoadajuvant therapy is unable to eliminate CTCs undergoing EMT [35].

In regard to lymph nodes involvement and CTCs detection rate, no clear association is apparent. Some studies show similar CTCs detection rate in LNM− and LNM+ breast cancer patients [7,32,33,35,59], which would indicate similar seeding potential of the tumor, but not similar colonization potential of the disseminated cells. However, preliminary results from a large SUCCESS trial revealed a correlation between CTCs presence and lymph nodes involvement [36] implying different seeding potential and possibly colonization potential. We have found significantly different CTCs detection rate (defined as *CK19+/MGB1+* or *CK19+/MGB1+/HER2+*) between LNM− and LNM+ patients (20% *vs.* 47%, *p* = 0.009 and 41% *vs.* 69%, *p* = 0.008, respectively), what supports the notion that tumors from LNM+ patients have superior seeding potential in comparison to LNM− patients. We would not have drawn the same conclusion if *CK19* was the only CTCs marker in our study, as in that case there was no significant difference in CTCs marker detection rate between LNM− and LNM+ patients (20% *vs.* 32%, *p* = 0.17). Also the study of Pecot [60] shows that cytokeratin expression should not be an ultimate marker for CTCs identification as cytokeratin-negative cancer cells can be found both in circulation and within primary tumors.

We are aware of limitations of our study, like the relatively small sample size and short follow-up period, which does not allow for assessing the impact of the analyzed markers on patients’ survival. However, it must be underlined that patients in this prospective study are still under observation and survival analysis will be done when data are mature. The method we applied—RT-qPCR—has many advantages, including high sensitivity and reproducibility, but it does not allow visualization of CTCs, and therefore, the detection of CTCs with EMT phenotype by this method is an indirect assessment. Obtained results are expressed as average gene expression obtained for the pool of cancer cells, so no heterogeneity within PT, LNM or individual CTCs can be analyzed. But the method has been carefully standardized [24]. Moreover, the results we present are strictly correlative in nature and present the general dependence between variables. Hence, the molecular characteristics of CTCs-EBF associated with lymph node involvement is a hypothesis generating finding, in line with recent publications [30]. Additional analyses aiming at understanding molecular mechanisms of the observed phenomenon are required to corroborate our hypothesis.

## 4. Experimental

### 4.1. Clinical Material

This study included 99 consecutive breast cancer patients treated at the Medical University Hospital in Gdansk between April 2011 and January 2013. Inclusion criteria were signed inform consent, primary operable breast cancer confirmed by histological examination and chemonaive status. Patients who received previous systemic treatment were not eligible for the study. Detailed characteristics of the studied group is presented in Table 8. ER and PgR were scored according to classical Allred system with cut-point 3 for positive result. HER2 positivity was based on standard criteria: 3+ in immunohistochemistry or positive result in fluorescence in situ hybridization (FISH), as previously described [61]. Median follow-up time was 1.6 years (0.2 to 2.6 years). To date, five deaths were observed, which is insufficient for performing survival analysis; however, follow-up data continue to be collected.

**Table 8:** Patients’ characteristics.

Variable	Number of cases (%)	
N	99	(100)
Age Median (range)	62 (33–85.5)	
T stage		
T1	36	(36)
T2	54	(55)
T3	3	(3)
T4	5	(5)
Tx	1	(1)
N stage		
N0	44	(44)
N1-3	55	(56)
Grade		
G1	13	(13)
G2	50	(51)
G3	35	(35)
Missing data	1	(1)
Histological type		
Ductal	75	(76)
Lobular	12	(12)
Other	11	(11)
Missing data	1	(1)
HER2 status *		
Negative	71	(72)
Positive	26	(26)
Missing data	2	(2)
HR status		
Negative	19	(19)
ER and/or PgR positive	80	(81)
Molecular subtype		
Luminal A	33	(33)
Luminal B (HER2-)	24	(24)
Luminal B (HER2+)	20	(20)
HER2+	6	(6)
Triple negative	13	(13)
Missing data	3	(3)

* HER2 positive status: 3+ in immunohistochemistry or positive in FISH test.

**Figure 2:**
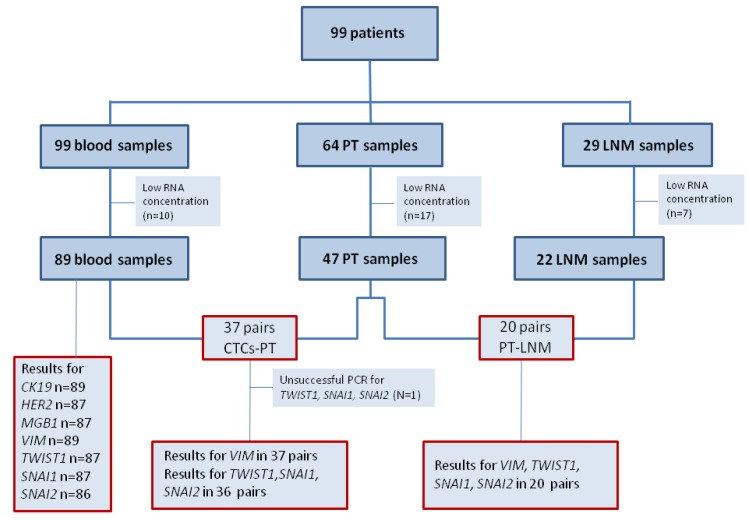
Flow chart of analyzed samples.

Abbreviations: PT—primary tumor, LNM—lymph node metastasis, n—number of cases.

Tumors were divided into five surrogate intrinsic subtypes [62] based on the expression of ER, PgR, HER2, tumor grade and/or Ki-67: (1) Luminal A—ER+ and/or PgR+, HER2−, Ki-67 below 14% or G1/2; (2) luminal B (HER2-negative)—ER+ and/or PgR+, HER2−, Ki-67 above 14% or G3; (3) luminal B (HER2-positive)—ER+ and/or PgR+, HER2+, any Ki-67 or any G; (4) HER2+—ER− and PgR−, HER2+; (5) Triple negative (TNBC)—ER−, PgR−, HER2−. From 99 breast cancer patients peripheral blood samples (N = 99), matched PT (N = 47) and LNM (N = 22) were collected (Figure 2).

Peripheral blood samples were drawn (5–10 mL) to the EDTA-coated tubes before tumor excision and systemic therapy initiation. To minimize possibility of keratinocytes contamination during skin punctuation, first few milliliters of blood were discarded. Samples were stored at 4 °C (for no longer than 24 h) until they were subjected to CTC-enrichment. Blood samples from 12 healthy women were similarly drawn and processed. 

PT and LNM removed during surgery were examined by a pathologist and subjected to formalin fixation (4% neutral-buffered formalin for up to 24 h) and paraffin embedding. Samples sections were stained with hematoxin-eosin to visualize primary tumors/lymph nodes metastases morphology, what allowed for selection of representative fragments containing invasive tumor component. Ten micrometer sections were cut and stored at 4 °C until RNA isolation. Additionally, sections of non-cancerous breast tissue and non-involved lymph nodes were collected. The study was conducted in accordance with the Declaration of Helsinki and approved by the local Ethical Committee of the Medical University of Gdansk.

### 4.2. RNA Extraction from Formalin-Fixed Paraffin-Embedded (FFPE) Tissue

Total RNA was isolated from 2–4 section of 10 µm thick FFPE tissues using RNeasy FFPE Kit (Qiagen, Hamburg, Germany) according to the manufacturer’s protocol. Samples were de-paraffinized by xylene wash followed by 100% ethanol wash. After the isolation RNA was digested using Turbo DNase Kit (Ambion, Austin, TX, USA) according to the manufacturer’s instruction (“Rigorous DNaze treatment” protocol) adding four units of DNaze per sample and digestion at 37 °C for 25 min.

### 4.3. Reverse Transcription

RNA (up to 10 µL) isolated from both CTC-enriched fractions and PT and LNM samples was reverse transcribed with Transcriptor cDNA First Strand Synthesis Kit (Roche, Basel, Switzerland) using random hexamers as primers. To monitor presence of contaminants that might influence both reverse transcription and qPCR 1 µL of external RNA molecule (RNA Spike from Solaris RNA Spike Control Kit, Thermo Scientific, Rockford, IL, USA) was added to each patient’s sample and control sample (containing water instead of isolated RNA thus carrying no inhibitors). cDNA samples were stored at −20 °C until gene expression analysis. Exogenous molecule was detected in qPCR, cycle thresholds (Cq) of control and patients’ samples were compared to assess the occurrence of inhibition (detection of RNA Spike molecule at later cycles indicated inhibition). 

### 4.4. Real-Time PCR (qPCR)

Gene expression analysis was performed using pre-design, commercially available assays containing primers and probes (TaqMan Gene Expression Assay, Applied Biosystems, Foster City, CA, USA) for detection of *TWIST1* (Hs00361186_m1, UniGene Hs.644998), *SNAI1* (Hs00195591_m1, UniGene Hs.48029), *SNAI2* (Hs00950344_m1, UniGene Hs.360174), *CK19* (*KRT19*, Hs01051611_gH; UniGene Hs.654568), *HER2* (Hs99999005_mH; UniGene Hs.446352), *MGB1* (*SCGB2A2*; Hs00935948_m1; UniGene Hs.46452) and reference genes *GAPDH* (Hs99999905_m1, UniGene Hs.544577) and *YWHAZ* (Hs03044281_g1; UniGene Hs.492407). Reference genes were chosen based on their expression stability (M parameter) in 10 samples of CTCs-EBF, 10 PT and 10 LNM assessed using geNorm applet (M parameter for *GAPDH*—0.395, 0.641 and 0.485 in CTCs-EBF, PT and LNM, respectively; M parameter for *YWHAZ*—0.500, 0.641 and 0.374 in CTCs-EBF, PT and LNM, respectively). TaqMan^®^ Universal PCR Master Mix (Applied Biosystems) was used, 4 µL of diluted cDNA was added per reaction, giving a total reaction volume of 20 µL. Reactions were performed in duplicate on 96-well plates in CFX96 thermal cycler (Bio-Rad, Hercules, CA, USA). Inter-run calibrator (cDNA obtained from healthy breast tissue) and no template controls were included in every plate. Moreover, in case of *YWHAZ* and *CK19* (the only assays that could detect also genomic DNA) genomic DNA contamination was monitor adding the same amount of untranscribed RNA to a PCR reaction. The samples was considered genomic DNA-free if the Cq difference between the sample containing RNA and cDNA was equal to or higher than 5.

Gene expression was calculated with a qBasePLUS software version 2.1 using a modified ∆∆Ct approach that corrects for a run-to-run variation. Gene expression levels were scaled to control samples—in the case of CTC-enriched samples the control samples were the samples with minimal expression level of each gene; in case of PT—healthy FFPE breast sample was chosen as a control, and for LNM non-involved FFPE lymph node. CTC-enriched blood samples were considered positive for expression of a given gene when the expression of that gene was higher than the highest expression in healthy control samples. For PT and LNM median value of gene expression was a cut-off value for positivity. For multimarker method, CTCs positivity was defined as positivity for at least one of the markers (*CK19*, *MGB1* or *HER2*).

### 4.5. Statistical Analysis

The patients’ characteristics were summarized using the median (range) for continuous variables and frequency (percentage) for categorical variables. Categorical variables were compared by Fisher’s exact test, and continuous variables were compared by the Spearman’s rank order test. Mann-Whitney test was used to examine correlations between quantitative values (gene expression level, age) and qualitative parameters (T stage, grade, LN status). Significance was defined as *p* < 0.05. STATISTICA software [63] was used for statistical analyses.

Concordance between PT and LNM or CTCs-EBF was measured by estimating Cohen’s kappa coefficient (κ) with Medcalc software [64]. The level of agreement based on κ values was assessed using the Landis and Koch criteria: 0.00–0.20, slight agreement; 0.21–0.40, fair agreement; 0.41−0.60, moderate agreement; 0.61–0.80, substantial agreement; and 0.81–1.00, almost perfect agreement [65].

## 5. Conclusions

PT shared more similarities with LNM than with CTCs-EBF, which might indicate that dissemination via the lymphatic route requires less extensive phenotypical/gene expression changes in tumor cells than hematogenous dissemination. LNM showed increased expression of EMT-related markers in comparison to PT, but EMT itself in PT did not seem to be necessary for lymphatic dissemination. The mesenchymal phenotype of CTCs-EBF correlated with poor clinicopathological characteristics of the patients. This study will be the basis for future evaluation of the outcome of the disease and the prognostic value of early-detected CTCs with mesenchymal phenotype. 
